# The Effectiveness of Catheter Ablation in the Management of Ventricular Tachycardia in Comparison With Antiarrhythmic Drugs in Patients With Structural Heart Disease: A Meta-Analysis

**DOI:** 10.7759/cureus.33608

**Published:** 2023-01-10

**Authors:** Jithin Karedath, Antonia Lisseth Valle Villatoro, Sana Faisal, Indu Kathuria Anand, Venkata Anirudh Chunchu, Muhammad Umer, Samprith Ala, Adil Amin

**Affiliations:** 1 Internal Medicine, James Cook University Hospital, Middlesbrough, GBR; 2 Medicine, University of Salvador, San Salvador, SLV; 3 Internal Medicine, California Institute of Behavioral Neurosciences & Psychology, Fairfield, USA; 4 Internal Medicine, Jinnah Sindh Medical University, Karachi, PAK; 5 Medicine, University of Delhi, Delhi, IND; 6 Medicine, Avalon University School of Medicine, Willemstad, CUW; 7 Internal Medicine, Dow University of Health Sciences, Civil Hospital Karachi, Karachi, PAK; 8 Cardiology, Pakistan Navy Station (PNS) Shifa, Karachi, PAK

**Keywords:** antiarrhythmic drugs, meta-analysis, structural heart disease, catheter ablation, ventricular tachycardia

## Abstract

The aim of this meta-analysis is to compare the safety and efficacy of catheter ablation versus antiarrhythmic drugs (AADs) in the management of ventricular tachycardia (VT) in patients with structural heart diseases. Two independent investigators searched electronic databases including PubMed, Cochrane, and Excerpta Medica database (EMBASE) using keyword combinations (Medical Subject Headings (MeSH) terms and free terms) such as “catheter ablation,” “ventricular tachycardia,” “escalation,” and “antiarrhythmic drugs” from inception to November 30, 2022. The primary efficacy outcomes included recurrence of VT at follow-up, all-cause mortality, and cardiovascular mortality. The secondary efficacy outcomes assessed in the current meta-analysis included implantable cardioverter-defibrillator (ICD) shock and hospitalization due to cardiac reasons. Safety outcomes included treatment-related adverse events and serious adverse events. A total of three studies were included in this meta-analysis. There was no significant difference in the risk of recurrence of VT (RR: 0.94, 95% CI: 0.72-1.24, p-value: 0.67), all-cause mortality (RR: 0.99, 95% CI: 0.67, 1.46, p-value: 0.98), cardiovascular mortality (risk ratio (RR): 0.90, 95% confidence interval (CI): 0.56-1.45, p-value: 0.67), incidence of ICD shocks (RR: 0.99, 95% CI: 0.76-1.29, p-value: 0.93, I-square: 0%), and hospitalization due to cardiac reasons in follow-up (RR: 0.77, 95% CI: 0.55-1.07, p-value: 0.12) between the catheter ablation group and the antiarrhythmic drug group. However, the risk of treatment-related adverse events was lower in the ablation group compared to the antiarrhythmic medicine (AAM) group (RR: 0.44, 95% CI: 0.29-0.67, p-value: 0.0001). In this meta-analysis of three randomized controlled trials (RCTs) among patients with structural heart disease who had ventricular tachycardia, the incidence of the recurrence of VT, all-cause mortality, cardiovascular mortality, and ICD shock was not significantly different between patients who received catheter ablation and antiarrhythmic drugs. However, regarding safety, catheter ablation is a safe procedure with a low risk of treatment-related events compared to antiarrhythmic drugs.

## Introduction and background

Ventricular tachycardia (VT) is an abnormal rhythm arising from the ventricles. It can occur in hearts that are either abnormal or normal in structure. VT is characterized by tachycardia with a wide QRS on an electrocardiogram (ECG). Ischemic heart disease (IHD) is one of the common causes of VT, and patients with IHD can present with monomorphic or polymorphic VT [1]. Polymorphic VT is commonly linked with acute myocardial ischemia. It is often managed with treatment to decrease ischemia including interventions for coronary revascularization (restoring myocardial blood supply) [2].

Practically, one of the immediate priorities in the acute management of patients with ongoing VT is sinus rhythm restoration. This is generally accomplished utilizing antiarrhythmic drugs (AADs) or electric direct current cardioversion with correction of any underlying precipitant of VT [3]. The goal then shifts to preventing future VT, and medication therapy is typically continued to lower this risk [2]. The commonly used antiarrhythmic drugs included ranolazine, dronedarone, dofetilide, sotalol, amiodarone, propafenone, mexiletine, quinidine, and disopyramide.

In general, individuals who report recurrent VT have two treatment options: catheter ablation or a change in their medication regimen, known as escalation of antiarrhythmic therapy [4]. Viably, escalation entails starting a new antiarrhythmic medication in addition to or in place of the medication they were taking when their recurrent VT first occurred. Increased recurrence of VT, unfavorable drug interactions, and/or side effects may prevent pharmacological therapy from progressing. In these situations, additional therapeutic escalation with a different AAD regime or VT ablation should be taken into consideration. Recurrent VT in individuals with an IHD may also lead to various therapies, such as shocks, which can have severe negative psychological impacts [4].

Catheter ablation is a well-known treatment for recurrent ventricular tachycardia (VT) in patients with ischemic heart disease (IHD) [5]. This minimally invasive treatment is performed in a cardiac catheter laboratory while the patient is sedated or given a general anesthetic agent. The purpose of ablation is to locate the portions of aberrant myocardium (i.e., scar) responsible for VT and make them electrically inert so they can no longer sustain it. Catheter ablation offers a therapeutic option for reducing episodes of VT in coronary artery disease (CAD) patients [6]. In the recent period, the role of catheter-based ablation has been growing in the short-term and long-term management of VT. This growth is mainly because of the significant enhancement in the understanding of the pathological basis of arrhythmias along with the continued improvement in technologies to ablate and identify the origin of VT [7].

Antiarrhythmic drugs are used as first-line treatment for patients with VT, but catheter ablation is gaining recognition as a vital option in recurrent VT. In current day-to-day practice, catheter ablation is considered a key strategy for patients with VT, when antiarrhythmic drugs are intolerable, ineffective, or not desired by patients. This meta-analysis aims to assess the effectiveness of catheter ablation in the management of VT in comparison with antiarrhythmic drugs in patients with structural heart disease.

## Review

Methodology

The current meta-analysis was reported according to the Preferred Reporting Items for Systematic Reviews and Meta-Analyses (PRISMA) guidelines.

Search Strategy and Study Selection

Two independent investigators searched electronic databases including PubMed, Cochrane, and Excerpta Medica database (EMBASE) using keyword combinations (Medical Subject Headings (MeSH) terms and free terms) such as “catheter ablation,” “ventricular tachycardia,” “escalation,” and “antiarrhythmic drugs” from inception to November 30, 2022. The reference lists of all included articles were also manually searched. All records have been imported to EndNote X7 Software. After removing duplicates, abstract and title screening were done by two authors independently. The full texts of eligible studies were screened for inclusion and exclusion criteria to identify studies comparing the effectiveness of catheter ablation for VT in comparison to antiarrhythmic drugs in patients with structural heart diseases. All potential discrepancies between the two authors were resolved via discussion.

Eligibility Criteria

We included all randomized controlled trials (RCTs) that have compared the efficacy and safety of catheter ablation and antiarrhythmic drugs for VT in patients with structural heart diseases. No restrictions were placed on the year of publication. We excluded studies that were observational, non-randomized trials or cross-over studies.

Outcomes

The primary efficacy outcomes included recurrence of VT at follow-up, all-cause mortality, and cardiovascular mortality. The secondary efficacy outcomes assessed in the current meta-analysis included implantable cardioverter-defibrillator (ICD) shock and hospitalization due to cardiac reasons. Safety outcomes included treatment-related adverse events and serious adverse events.

Data Extraction

All data were extracted independently by two authors from the study text, tables, and figures using a predesigned form on Microsoft Excel (Microsoft Corp., Redmond, WA, USA). We extracted study and participants’ characteristics including author name, publication year, study setting, study groups, sample size, follow-up duration, and patient age and gender. All potential discrepancies between the two authors were resolved via discussion.

Risk of Bias Assessment

The risk of bias for each RCT was assessed by two investigators independently using the Cochrane Risk of Bias Tool. This tool covers six domains including selection bias, performance bias, detection bias, attrition bias, reporting bias, and other bias. Any disagreement between the two authors was resolved via consensus or discussion with the third investigator.

Statistical Analysis

All analysis was performed using the RevMan version 5.4.0 (Cochrane Collaboration, Software Update, Oxford, UK), and the risk ratio (RR) was calculated along with 95% confidence interval (CI) using the Mantel-Haenszel model. I-square statistic was used to calculate the proportion of overall study variation that can be attributed to heterogeneity rather than chance; values more than 50% are indicative of significant heterogeneity. In case of heterogeneity of more than 50%, the random effects model was used. Otherwise, the fixed effects model was used. P values were two-sided, and values of 0.05 were considered statistically significant. We did not assess the small study effect including publication bias, considering less than 10 eligible RCTs were identified.

Results

**Figure 1:**
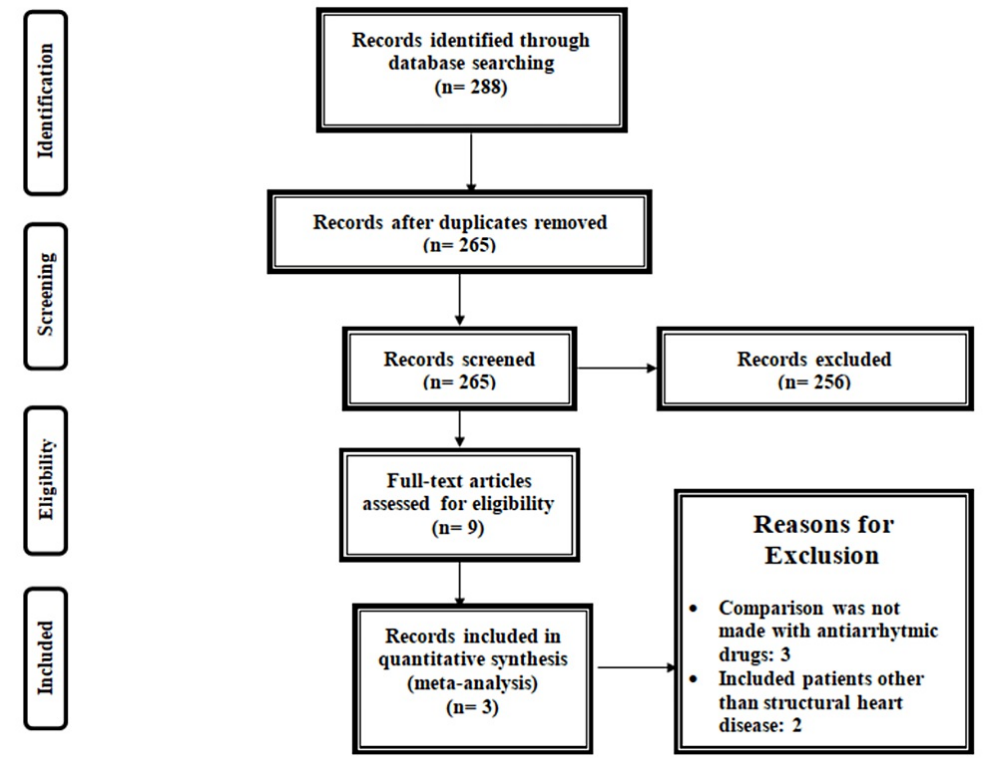
PRISMA flowchart of the selection of studies PRISMA: Preferred Reporting Items for Systematic Reviews and Meta-Analyses

**Figure 2:**
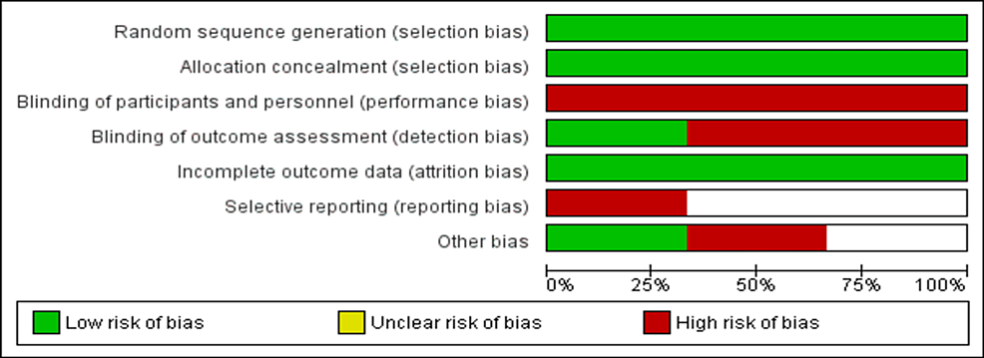
Graph of risk of bias assessment

Figure 1 shows the PRISMA flowchart of the selection of studies. A total of 288 studies were identified through online database searching. After removing duplicates, the titles and abstracts of 265 records were screened to assess whether they are eligible for full-text screening. A total of nine studies were selected for full-text screening, and three studies fulfilled the inclusion criteria and were included in the current meta-analysis. Table 1 shows the characteristics of the included studies. Out of three studies, two were multicenter, while one was conducted in a single center only. Follow-up of included studies ranged from six months to 48 months. The majority of participants in all included studies were males. Figure 2 shows the risk of bias assessment.

**Table 1:** Characteristics of the included studies

Author name	Year	Setting	Groups	Sample size	Follow-up duration	Mean age	Males (%)
Al-Khatib et al. [8]	2014	Multicenter	Catheter ablation	13	6 months	64.5 years	92.59%
			Antiarrhythmic drugs	14			
Arenal et al. [9]	2022	Single center	Catheter ablation	71	24 months	76 years	96%
			Antiarrhythmic drugs	73			
Sapp et al. [10]	2016	Multicenter	Catheter ablation	132	48 months	68.5 years	93.05%
			Antiarrhythmic drugs	127			

Primary Efficacy Outcomes

**Figure 3:**
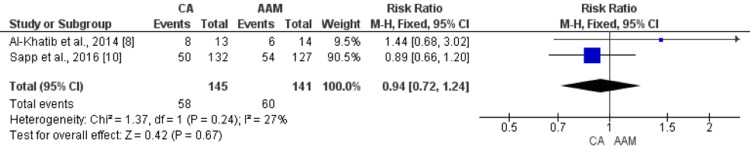
Forest plot comparing the effect of CA and AAM on the recurrence of ventricular tachycardia Sources: References [8,10] CA: catheter ablation, AAM: antiarrhythmic medicines, CI: confidence interval

Two studies have compared the rate of recurrence of VT between the ablation arm and the antiarrhythmic medicine (AAM) arm. The pooled incidence of the recurrence of VT was 40% in the ablation group and 42.55% in the control group. Although the incidence of VT was lower in the ablation group, the difference is statistically insignificant (RR: 0.94, 95% CI: 0.72-1.24, p-value: 0.67), as shown in Figure 3. No significant heterogeneity was reported among the study results (I-square: 27%, p-value: 0.24).

**Figure 4:**
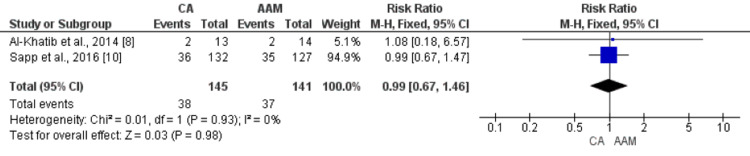
Forest plot comparing the effect of CA and AAM on all-cause mortality Sources: References [8,10] CA: catheter ablation, AAM: antiarrhythmic medicines, CI: confidence interval

**Figure 5:**
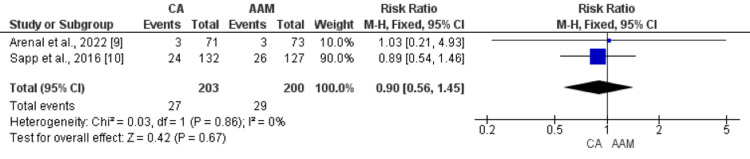
Forest plot comparing the effect of CA and AAM on cardiac-related mortality Sources: References [9,10] CA: catheter ablation, AAM: antiarrhythmic medicines, CI: confidence interval

Two studies compared the risk of all-cause mortality between patients in the ablation group and the AAM group. Upon meta-analysis, no statistically significant difference was found in all-cause mortality between patients who were randomized to the ablation group and patients who were randomized to the AAM group (RR: 0.99, 95% CI: 0.67, 1.46, p-value: 0.98), as shown in Figure 4. No heterogeneity was found among the study results (I-square: 0%, p-value: 0.93). Similarly, no significant difference was found between the two groups in terms of cardiovascular mortality (RR: 0.90, 95% CI: 0.56-1.45, p-value: 0.67), as shown in Figure 5.

Secondary Efficacy Outcomes

**Figure 6:**
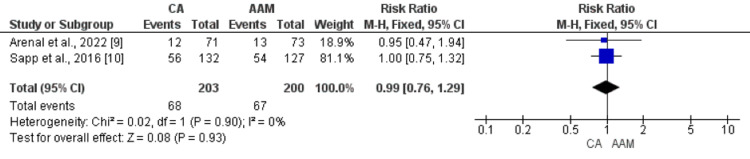
Forest plot comparing the effect of CA and AAM on ICD shock Sources: References [9,10] CA: catheter ablation, AAM: antiarrhythmic medicines, CI: confidence interval

**Figure 7:**
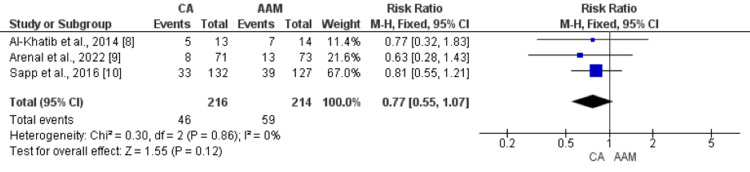
Forest plot comparing the effect of CA and AAM on hospitalization Sources: References [8-10] CA: catheter ablation, AAM: antiarrhythmic medicines, CI: confidence interval

The incidence of ICD shocks was reported in two RCTs. No significant difference was found between the ablation group (33.49%) and the AAM arm (33.50%) (RR: 0.99, 95% CI: 0.76-1.29, p-value: 0.93, I-square: 0%), as shown in Figure 6. Hospitalization due to cardiac reasons occurred in 46 of 216 (21.30%) patients in the ablation group compared to 59 of 214 (27.57%) in the AAM group. No significant difference was found between the two groups in terms of the incidence of hospitalization due to cardiac reasons in follow-up (RR: 0.77, 95% CI: 0.55-1.07, p-value: 0.12, I-square: 0%), as shown in Figure 7.

Safety Outcomes

No significant difference in the incidence of serious adverse events was found between the two study groups upon meta-analysis of the three studies (RR: 1.03, 95% CI: 0.77-1.39, p-value: 0.85, I-square: 0%). Regarding the risk of treatment-related adverse events, the risk was significantly lower in patients randomized in the ablation group compared to the AAM group (RR: 0.44, 95% CI: 0.29-0.67, p-value: 0.0001, I-square: 0%).

Discussion

In this meta-analysis of three RCTs, no significant difference was found between the two groups in terms of any of the efficacy outcomes reported in the current meta-analysis. However, it is found that the risk of treatment-related adverse events is significantly lower in the catheter ablation group compared to patients randomized in the antiarrhythmic group.

In practice, the choice between catheter ablation on one hand versus starting or changing antiarrhythmic drug therapy on the other hand is often the most pertinent decision to be taken. This question was addressed by three of the included clinical trials. However, the results are highly insignificant because of the lower sample size. This is the first meta-analysis conducted to compare the effectiveness of catheter ablation in the management of patients with recurrent VT in comparison to antiarrhythmic drugs in patients with structural heart disease. The previous meta-analysis was conducted to compare the clinical outcomes of catheter ablation for VT versus medical therapy [11,12]. These studies supported catheter ablation in terms of reducing the recurrence of VT and reported fewer adverse effects in the catheter ablation group compared to medical therapy. However, our meta-analysis is different from past meta-analyses as it has compared catheter ablation with antiarrhythmic drugs rather than all medical therapies for the management of VT.

The current meta-analysis showed that neither of the two treatments showed superiority with respect to all-cause mortality, risk of ICD shock, cardiovascular mortality, and recurrence of VT. The benefit with respect to the secondary outcome of ablation was driven by a reduction in the rates of hospital admission due to cardiovascular reasons. Hospital admissions due to cardiac reasons were higher in the escalated therapy group, but the difference was insignificant. However, adverse event-related treatments were more frequent in patients receiving antiarrhythmic drug compared to patient receiving catheter ablation.

The guidelines and consensus recommended the utilization of catheter ablation if antiarrhythmic drugs do not prevent recurrent VT [13,14]. However, these guidelines are largely based on case series and expert opinions. The current meta-analysis provides some evidence that catheter ablation needs to be preferred over antiarrhythmic therapy to reduce VT recurrence in patients with structural heart disease. It is important to keep in mind that ventricular arrhythmias cannot be cured by either of the conventional therapies for VT, which is antiarrhythmic medications (AAMs) plus ICD installation [15]. To suppress VT, long-term AAD treatment may be required; nevertheless, serious side effects should be considered, especially in patients with numerous comorbidities including pulmonary, renal, or hepatic illnesses [16]. For patients with a history of myocardial infarction and repeated bouts of symptomatic sustained VT, who present with VT or VF and have tried and failed to respond to AADs, or who are intolerable to AADs, current guidelines advise catheter ablation [17].

The current meta-analysis has certain limitations. Firstly, it is composed of only three studies, and the primary outcomes were not assessed by each of the studies included in the current meta-analysis, resulting in low statistical power. Secondly, due to a low number of studies, we were not able to assess publication bias. Moreover, catheter ablations performed in the studies were done in expert centers by experienced doctors committed to a comprehensive approach, while the findings may not be generalizable to less experienced settings. The majority of the trials evaluated the primary outcome using ICD arrhythmic logs; however, the programming parameters varied between studies, which could have affected the findings. It is necessary to predefine standard zones while organizing upcoming trials.

## Conclusions

In this meta-analysis of three RCTs among patients with structural heart disease who had ventricular tachycardia, the incidence of the recurrence of VT, all-cause mortality, cardiovascular mortality, and ICD shock was not significantly different between patients who received catheter ablation and antiarrhythmic drugs. However, regarding safety, catheter ablation is a safe procedure with a low risk of treatment-related events compared to antiarrhythmic drugs. It is evident from the findings of this meta-analysis that catheter ablation is a safe procedure as lower adverse events were reported in patients receiving catheter ablation compared to patients receiving antiarrhythmic drugs. However, the findings of this meta-analysis need to be interpreted with caution because of the low sample size in each of the outcomes. In the future, more prospective randomized trials need to be conducted enrolling a large population to guide professionals so more robust recommendations can be developed for the management of patients with ventricular tachycardia.
